# The Inhibitory Effects of *Cordyceps militaris* ARA301 Extract on Lipopolysaccharide-Induced Lung Injury *in vivo*

**DOI:** 10.4014/jmb.2412.12043

**Published:** 2025-03-06

**Authors:** Hye Kang Seong, Min Jeong Kim, Ardina Nur Fauziah, Hyeon Su Jeong, Hyo Jeong Kim, Chung Yeol Yang, Su Jin Park, Soo Yeon Bae, Sung Keun Jung

**Affiliations:** 1 School of Food Science and Biotechnology, Kyungpook National University, Daegu 41566, Republic of Korea; 2 Bioara Co., Ltd., Seoul 08375, Republic of Korea; 3 Tailored Food Technology, Kyungpook National University, Daegu 41566, Republic of Korea

**Keywords:** Health functional food, respiratory health, *Cordyceps militaris*, ARA301, inflammation, bronchoalveolar lavage fluid

## Abstract

Lung injury is a critical health concern associated with severe inflammatory responses and tissue damage that can weaken respiratory function and potentially become life-threatening in severe cases. This study aimed to establish a mouse model of lung injury induced by lipopolysaccharides (LPS) derived from *Klebsiella pneumoniae* and to evaluate whether *Cordyceps militaris* ARA301 extract (CME) can prevent lung injury. CME was orally administered to mice for three consecutive days, followed by intranasal LPS administration. Mice were sacrificed 24 h later to analyze immune cell alterations and inflammatory responses through bronchoalveolar lavage fluid (BALF) and tissue analyses. CME administration inhibited immune cell infiltration, tissue fibrosis, and excessive mucus deposition induced by intranasal LPS administration. Furthermore, CME suppressed the expression of mucin 5AC (MUC5AC), a protein involved in mucus production, as well as the expression of inducible nitric oxide synthase (iNOS), and cyclooxygenase-2 (COX-2) in lung tissues. In BALF, CME reduced the production of interleukin-1 beta (IL-1β), interleukin-6 (IL-6), tumor necrosis factor-alpha (TNF-α), and C-X-C motif chemokine ligand 1 (CXCL1), which were elevated due to LPS administration. Additionally, CME decreased the total immune cell, neutrophil, monocyte, and eosinophil numbers in BALF. The anti-inflammatory activity of CME was evaluated *in vitro* using RAW 264.7 cells. CME treatment reduced the secretion of pro-inflammatory cytokines induced by LPS and inhibited the phosphorylation of p65, inhibitor of kappa B alpha (IκBα), and IκB kinase alpha (IKKα). These findings suggest that CME has potential as a functional health supplement effective in preventing lung injury.

## Introduction

Respiratory diseases pose a significant global health burden. Chronic obstructive pulmonary disease (COPD) is the fourth leading cause of death, responsible for approximately 5% of global mortality [1]. The coronavirus disease 2019 (COVID-19) pandemic has further intensified interest in respiratory health, underscoring the need for innovative preventive approaches. However, nutraceuticals and pharmaceuticals aimed at improving respiratory health remain underdeveloped in many regions, including South Korea. This highlights the necessity of exploring novel bioactive materials for respiratory disease prevention.

Damage to lung tissue caused by external stimuli including pathogens and environmental pollutants or internal inflammatory responses results in impaired respiratory function [2]. Given the Lungs' critical role in oxygen-carbon dioxide exchange, such injuries can have systemic consequences, extending beyond respiratory dysfunction. Respiratory diseases associated with lung injury such as COPD and acute lung injury (ALI) are driven by complex interactions of physiological and molecular mechanisms [3]. Pathogens including *Klebsiella pneumoniae*, *Streptococcus pneumoniae*, and *Haemophilus influenzae* exacerbate these diseases and are commonly found in patients with compromised respiratory health [4-6].

The progression of respiratory diseases involves immune cell infiltration with abnormal production of chemokines and inflammatory mediators which exacerbate tissue damage [7]. Neutrophils, key players in the innate immune response, migrate rapidly to inflammation sites but can cause damage through prolonged activation [8, 9]. The nuclear factor kappa B (NF-κB) pathway is a pivotal regulator of inflammation, controlling the transcription of inflammatory cytokines such as interleukin (IL)-1β, IL-6, and tumor necrosis factor (TNF)-α Additionally, inflammatory mediators and excessive neutrophil recruitment can activate this pathway [10]. Activation of the NF-κB pathway exacerbates respiratory dysfunction through tissue remodeling, fibrosis, and excessive mucus secretion [11, 12].

Cordyceps, a traditional medicinal fungus, has been valued for its diverse bioactive properties, including anti-inflammatory, antioxidant, and immune-modulatory effects [13]. *Cordyceps militaris* ARA301 extract (CME) is a novel strain enriched in cordycepin, a compound with proven anti-inflammatory potential. However, scientific evidence for the anti-inflammatory and respiratory health-improving effects of the strain is still needed.

This study investigates the protective effects of CME in a lipopolysaccharide (LPS)-induced lung injury model, evaluating its ability to suppress neutrophil activation, reduce inflammatory mediator secretion, and mitigate structural and functional lung damage. Additionally, its anti-inflammatory activity was assessed *in vitro* using RAW 264.7 macrophages. These findings aim to provide a foundation for CME as a functional food material for respiratory disease prevention.

## Materials and Methods

### Materials and Reagents

Dulbecco’s Modified Eagle’s Medium (DMEM), fetal bovine serum (FBS), and penicillin/streptomycin solution were obtained from Hyclone, USA). LPS from *K. pneumoniae*, 3-(4,5-dimethylthiazol-2-yl)-2,5-diphenyltetrazolium bromide (MTT), and dimethyl sulfoxide (DMSO) were purchased from Sigma Aldrich (USA). Primary antibodies, including inducible nitric oxide synthase (iNOS), mucin 5AC (MUC5AC), p65, phosphorylated p65 (p-p65), inhibitor of kappa B alpha (IκBα), phosphorylated IκBα (p-IκBα), IκB kinase alpha (IKKα) and phosphorylated IKKα (p-IKKα/β), were acquired from Cell Signaling Technology (USA) and cyclooxygenase-2 (COX-2) and β-actin primary antibodies were purchased from Santa Cruz (USA). Secondary antibodies were supplied by Thermo Fisher Scientific (USA).

### *Cordyceps militaris* ARA301 Extract Preparation

The CME was provided by BioAra Co., Ltd., registered under Plant Variety Protection No. 7041. *C. militaris* was cultivated on an insect-based medium for 60 days. The extract was obtained via hot-water extraction (80°C for 24 h), filtered, lyophilized, and dissolved in DMSO (100 mg/mL) for *in vitro* studies or sterile water for *in vivo* studies at concentrations of 100 mg/kg and 500 mg/kg.

### Animals and Experimental Design

Male C57BL/6 mice (7 weeks old) were obtained from Jabio (Republic of Korea) and housed under controlled conditions (23 ± 2°C, 12-h light/dark cycle) with ad libitum access to food and water. Animal experiments were approved by the Animal Care and Use Committee of Kyungpook National University (approval number KNU 2023-0433) and conducted according to their guidelines. Mice were acclimatized for 8 days before randomization into four groups: (1) control, (2) LPS, (3) CME low-dose (100 mg/kg), and (4) CME high-dose (500 mg/kg) (*n* = 10 per group). CME or sterile water was orally administered for three consecutive days. One hour after the final dose, all mice except those in the control group received an intranasal administration of LPS (50 μg/mouse). Control mice were administered sterile phosphate-buffered saline (PBS). Mice were sacrificed 24 h post-intranasal LPS administration for subsequent analyses.

### Histological Analysis

Lung tissues were harvested, fixed, and sectioned for hematoxylin and eosin (H&E), Alcian blue-periodic acid Schiff (AB-PAS), and Masson’s trichrome staining. Standard protocols were followed to assess immune cell infiltration, mucus deposition, and fibrosis. Slides were analyzed under a microscope (Leica Microsystems, Germany) using the Leica Application Suite X software (Leica Microsystems).

### Immunofluorescence

Frozen lung sections (10 μm) were prepared using a cryostat (CM1850, Leica Biosystems) and fixed with 4%formaldehyde. Sections were blocked with 5% FBS and 0.3% Triton X-100 and incubated with primary antibodies (iNOS 1:800, COX-2 1:100, and MUC5AC 1:400) overnight at 4°C. Secondary antibodies conjugated with Alexa Fluor 488 were applied, and nuclei were counterstained with DAPI. Fluorescence was visualized using a Leica microscope and quantified using ImageJ software.

### BALF Collection

BALF was collected by inserting a catheter into the trachea and washing the lungs with 1 ml sterile PBS. Samples were centrifuged at 450 ×*g* for 10 min at 4°C to separate supernatant and cell pellets for cytokine analysis and immune cell counting.

### ELISA for Cytokine and Chemokine Analysis

The levels of cytokines (IL-1β, IL-6, and TNF-α) and chemokines (CXCL1) in BALF were quantified using enzyme-linked immunosorbent assay (ELISA) kits (Thermo Fisher Scientific). All procedures followed the manufacturer's instructions.

### Immune Cell Analysis

BALF cell pellets were treated with red blood cell (RBC) lysis buffer and resuspended in PBS for flow cytometry. Cells were stained with antibodies against CD45, Ly6G, CD11b, and Siglec-F and analyzed using a Beckman Coulter flow cytometer. Wright’s staining was used to confirm neutrophil morphology.

### Cell Culture and Cell Viability

RAW 264.7 cells were cultured in DMEM supplemented with 10% FBS and antibiotics in a humidified incubator (37°C, 5% CO_2_). For viability assays, cells were treated with CME (25, 50, 100 μg/ml) for 24 h. Cytotoxicity was assessed using the MTT assay, and absorbance was measured at 595 nm using a SpectraMax iD3 microplate reader.

### Quantification of mRNA Using qRT-PCR

RAW 264.7 cells were treated with CME (25, 50, 100 μg/ml) for 1 h before LPS (1 μg/ml) stimulation for 6 h. Total RNA was extracted using RNAiso Plus and converted to cDNA. Quantitative PCR was performed using SYBR Green Real-Time PCR Master Mix (Toyobo Co., Ltd., Japan) with glyceraldehyde-3-phosphate dehydrogenase (GAPDH) as the reference gene. The primer sequences are displayed in Table 1. The comparative ΔΔCq method was used with CFX Maestro Software (Bio-Rad Inc., USA).

### Western Blot Assay

The lysed cells were vortexed for 30 min and centrifuged at 13,572 ×*g* for 15 min at 4°C to collect the supernatant. Protein concentration was determined using a detergent-compatible protein assay (Bio-Rad Laboratories). The prepared samples were loaded onto a 10% sodium dodecyl sulfate-polyacrylamide gel, and electrophoresis was performed. Proteins were then transferred to a polyvinylidene difluoride membrane (Immobilon-FL transfer membrane, Millipore, USA). The membrane was blocked with 5% skim milk, followed by incubation with a primary antibody diluted 1:1000 at 4°C overnight. After the reaction, the membrane was washed three times with Tris-buffered saline with Tween 20 (TBST) and then incubated with goat anti-rabbit IgG (H+L) or goat anti-mouse IgG (H+L) secondary antibody at room temperature for 1 h with shaking at 8 rpm. Finally, protein expression was detected using EzWestLumi plus (Dawinbio, Republic of Korea) and a GeneGnome XRQ-NPC Imager (Syngene, UK).

### Statistical Analysis

Data are presented as mean ± standard deviation (SD) from at least three independent experiments. Statistical significance was demonstrated by one-way analysis of variance (ANOVA) followed by Dunnett's post-hoc test, and a *p*-value < 0.05 was considered significant.

## Results

### Oral Administration of CME Inhibits LPS-Induced Pathological Damage in Mouse Lung Tissue

**Fig. 1:**
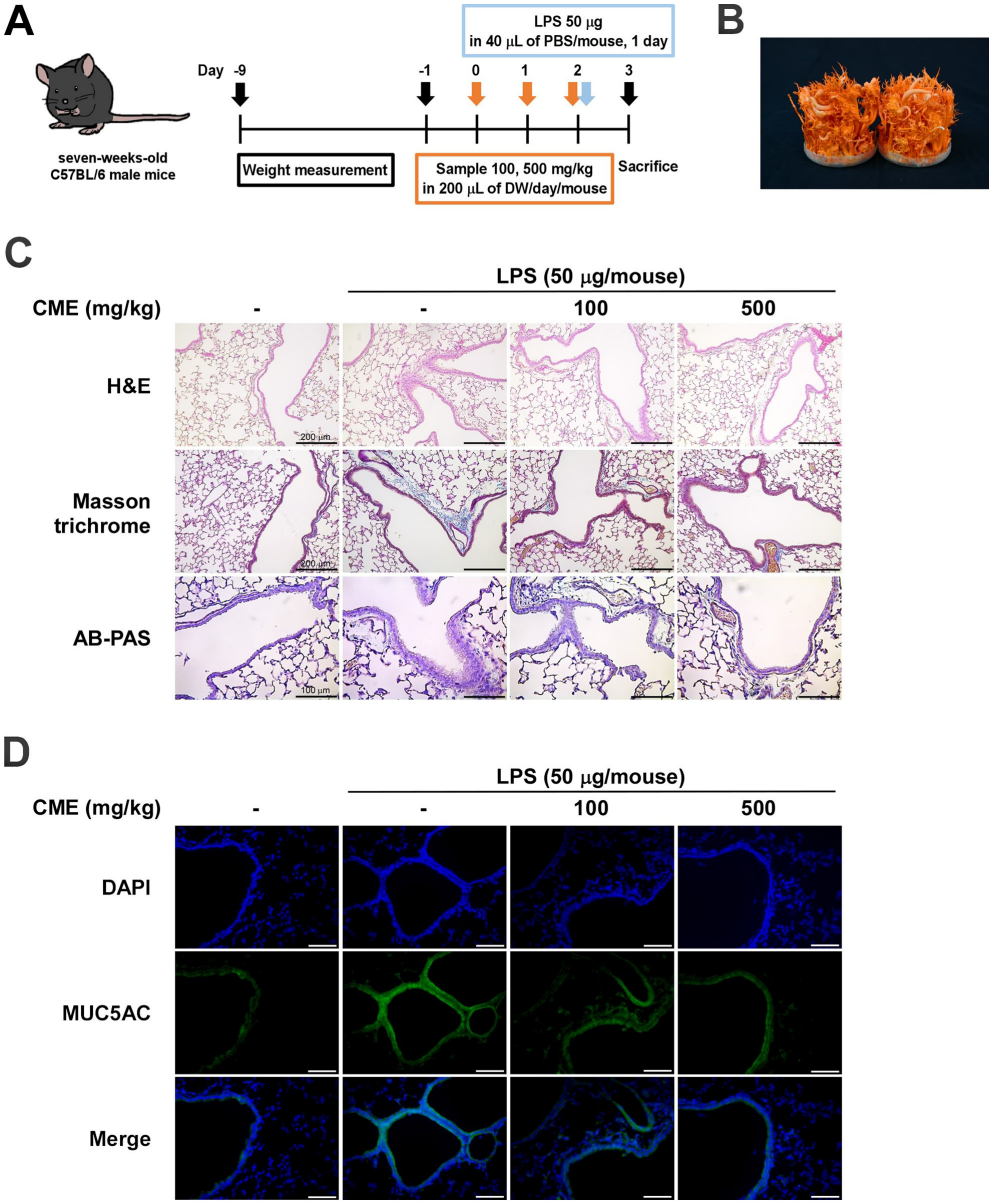
Oral administration of *Cordyceps militaris* ARA301 extract (CME) reduces LPS-induced pathological changes in lung tissue. (**A**) Schematic representation of the experimental design, including oral CME administration and LPS-induced lung injury model. (**B**) Image of *C. militaris* ARA301 used for extract preparation. (**C**) Representative histological images: H&E staining shows reduced immune cell infiltration; Masson’s trichrome staining demonstrates decreased collagen deposition (fibrosis); AB-PAS staining reveals reduced mucus secretion in CME-treated groups compared to the LPS-only group. (**D**) Immunofluorescence staining shows reduced expression of mucin 5AC (MUC5AC) in lung tissue of CME-treated mice.

Because *K. pneumoniae* is a bacterium that can infect the lungs and cause bacterial pneumonia [14], we used this LPS strain to provoke acute lung injury. We administered CME orally for 3 days and intranasally administered LPS from *K. pneumoniae* on the third day, and then evaluated the effect of CME on ALI through histological and biochemical analyses 24 h later (Fig. 1A). H&E staining revealed substantial immune cell infiltration and irregular tissue morphology in the LPS-treated group compared to the control group. These pathological changes were significantly alleviated in mice pretreated with CME (Fig. 1C). Masson’s trichrome staining indicated increased collagen deposition and pulmonary fibrosis in LPS-treated mice. In contrast, CME administration significantly reduced collagen deposition, suggesting inhibition of fibrosis (Fig. 1C). Similarly, AB-PAS staining showed increased mucus secretion in LPS-treated mice, which was suppressed by CME treatment (Fig. 1C). Immunofluorescence analysis revealed elevated expression of MUC5AC, a critical mediator of airway mucus secretion, in the LPS-treated group. CME administration significantly reduced MUC5AC expression, indicating reduced mucus hypersecretion (Fig. 1D).

### Oral Administration of CME Inhibits LPS-Induced Expression of iNOS and COX-2 in the Mouse Lung Tissue

**Fig. 2:**
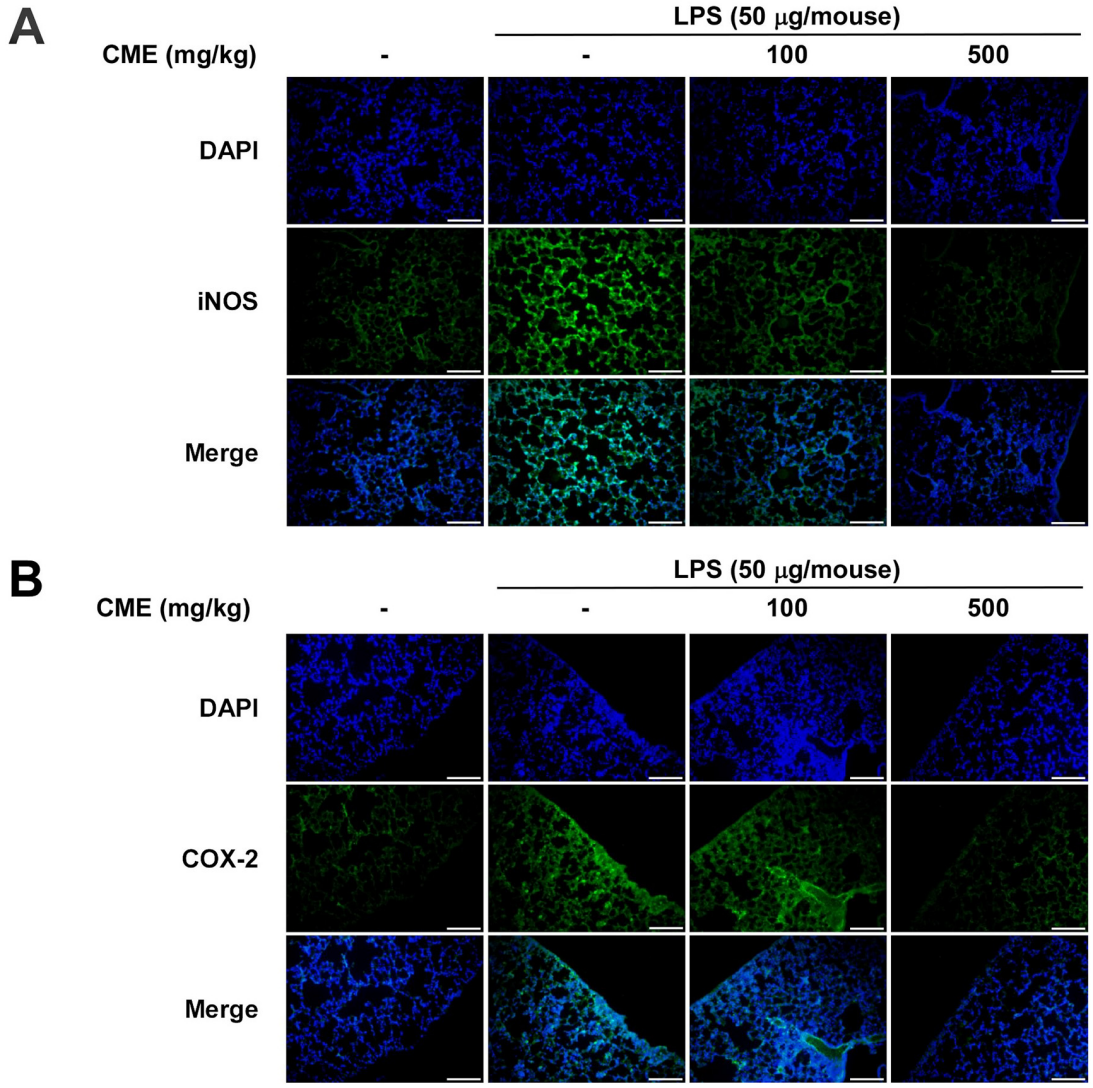
Oral administration of CME inhibits LPS-induced expression of iNOS and COX-2 in lung tissue. (**A**) Immunofluorescence staining demonstrates increased iNOS expression in the LPS-only group, which is significantly suppressed by CME pretreatment. (**B**) COX-2 expression, elevated in the LPS group, is reduced following CME treatment.

The expression of iNOS and COX-2, key enzymes in inflammatory responses, was assessed using immunofluorescence [15]. Intranasal administration of LPS markedly increased iNOS and COX-2 expression in lung tissue compared to controls. Oral administration of CME significantly suppressed the expression of iNOS and COX-2, highlighting its anti-inflammatory potential (Fig. 2A and 2B).

### Oral Administration of CME Suppresses the LPS-Induced Cytokine and Chemokine Levels in BALF

**Fig. 3:**
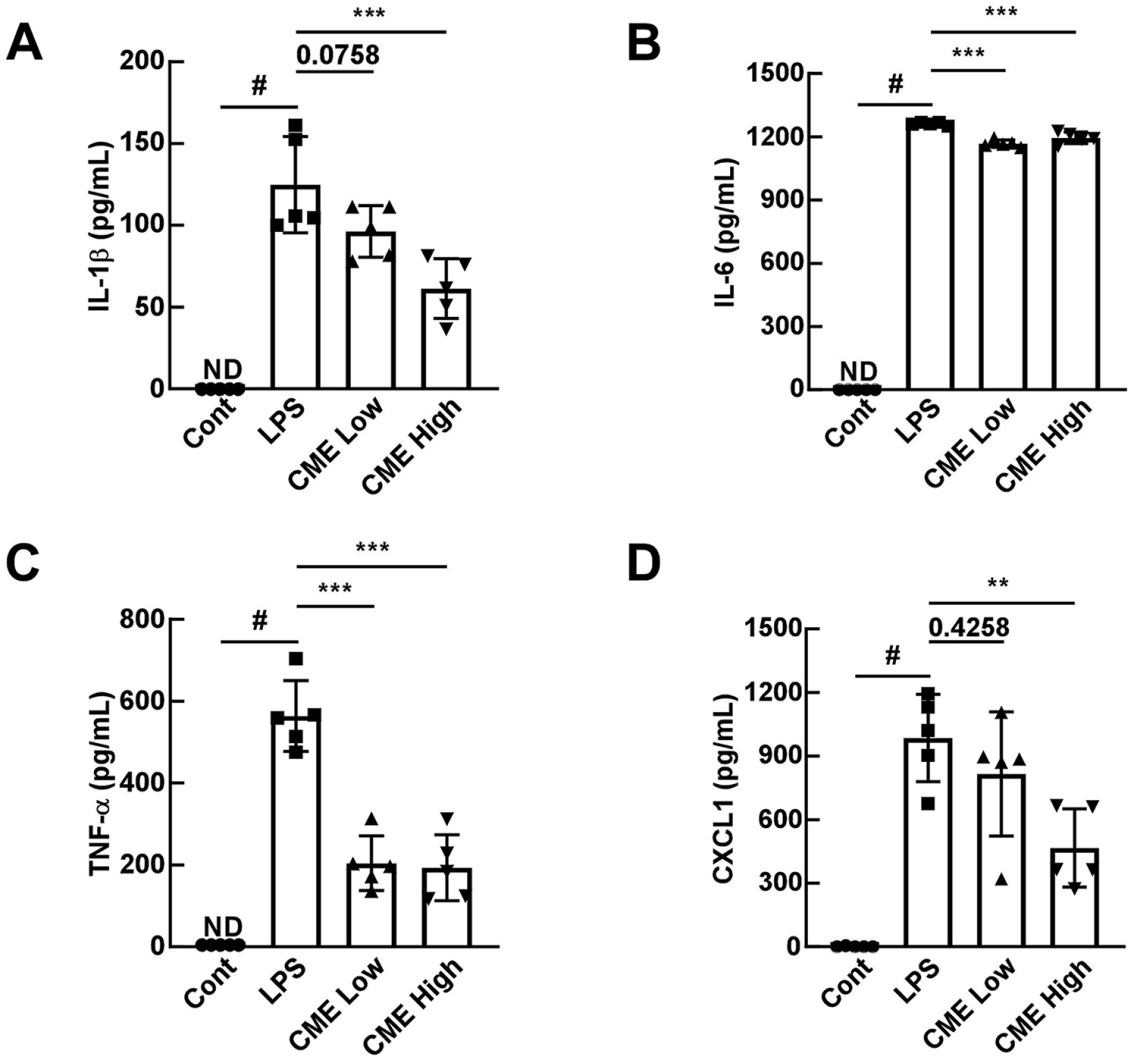
Oral administration of CME suppresses LPS-induced inflammatory cytokines and chemokines in BALF. (**A**–**D**) ELISA results show significant reductions in cytokine (IL-1β, IL-6, TNF-α) and chemokine (CXCL1) levels in BALF of CME-treated mice. Data are presented as mean ± SD. #*p* < 0.05 for the LPS induction level; **p < 0.01, ***p < 0.001 compared to the LPS-only group.

Exposure of the respiratory tract to pathogens including LPS causes abnormal immune response and subsequent acute inflammation, resulting in elevated levels of pro-inflammatory cytokines and chemokines such as IL-1β, IL-6, TNF-α, and CXCL1 in BALF [16]. The mean IL-1β expression level in BALF was 0 pg/mL in the control group, 124.8 pg/mL in the LPS group, 96.2 pg/mL in the low group, and 61.3 pg/mL in the high group. The mean IL-6 expression level was 0 pg/mL in the control group, 1261.6 pg/mL in the LPS group, 1167.5 pg/mL in the low group, and 1195.2 pg/mL in the high group. The mean TNF-α level was 4.9 pg/mL in the control group, 563.9 pg/mL in the LPS group, 204.2 pg/mL in the low group, and 193.3 pg/mL in the high group. The mean CXCL1 level was 1.0 pg/mL in the control group, 985.1 pg/mL in the LPS group, 815.5 pg/mL in the low group, and 466.1 pg/mL in the high group. CME administration significantly reduced the expression of these markers compared to the LPS-only administered group (Fig. 3A-3D). These findings suggest that CME mitigates inflammation by inhibiting cytokine and chemokine production.

### Oral Administration of CME Inhibits the Increase in LPS-Induced Immune Cell Number in the BALF of Mice

**Fig. 4:**
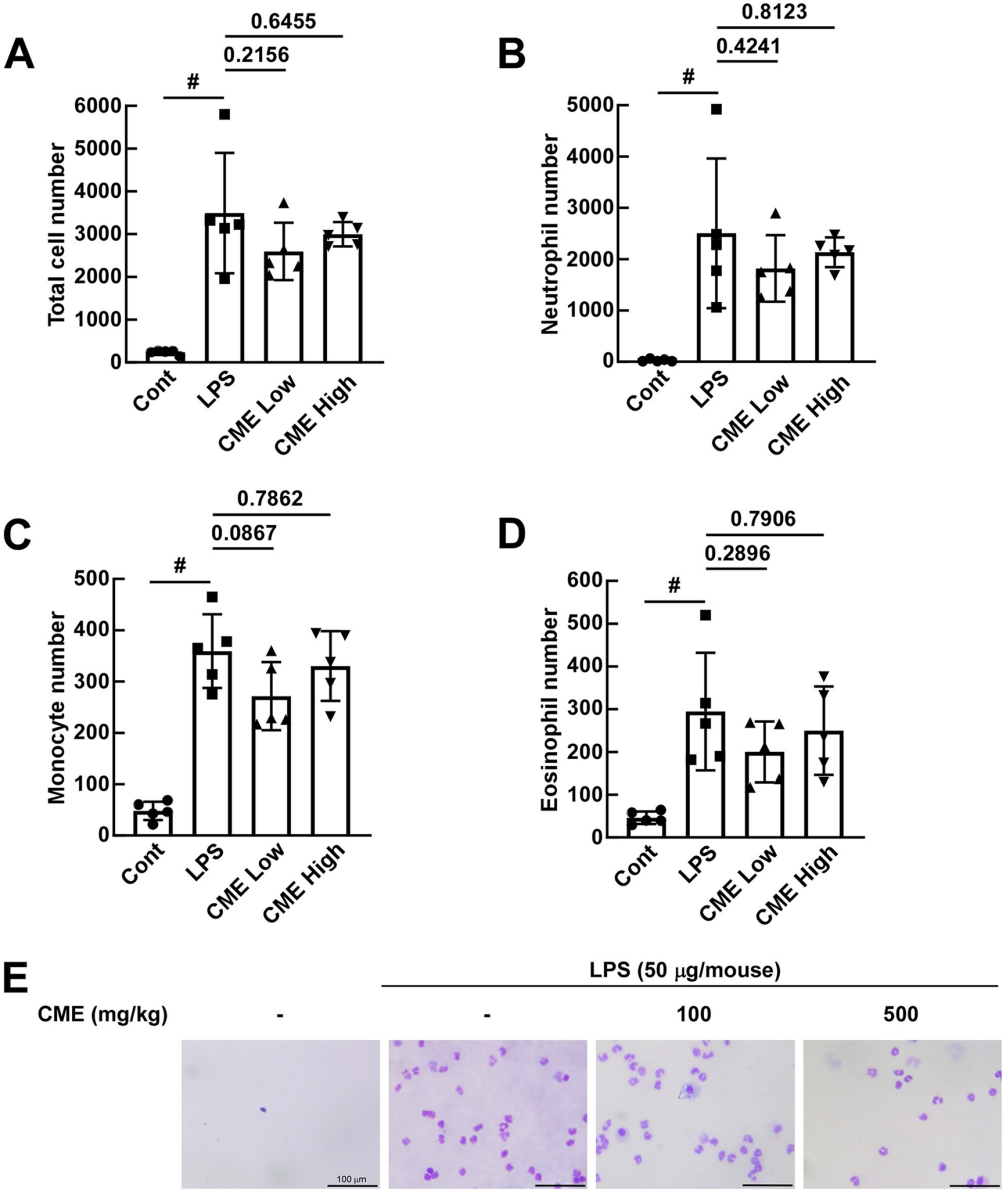
Oral administration of CME reduces immune cell infiltration and neutrophil recruitment in BALF. (**A**–**D**) Flow cytometry analysis shows decreased total immune cell, neutrophil, monocyte, and eosinophil numbers in BALF of CME-treated mice. (**E**) Wright’s staining reveals reduced neutrophil numbers in the CME-treated group compared to the LPSonly group. Data are presented as mean ± SD. #*p* < 0.05 for the LPS induction level.

Flow cytometry analysis of BALF revealed that the average total cell number was 235.6 in the control group, 3489.8 in the LPS group, 2595.0 in the low group, and 2996.8 in the high group. The average neutrophil number was 29.8 in the control group, 2502.2 in the LPS group, 1820.0 in the low group, and 2134.2 in the high group. The average monocyte number was 48.0 in the control group, 359.4 in the LPS group, 271.6 in the low group, and 330.2 in the high group. The average eosinophil number was 46.0 in the control group, 294.6 in the LPS group, 200.2 in the low group, and 249.8 in the high group. Statistical analysis indicated a significant increase in immune cell number in the LPS group compared to the control group. A decreasing trend in immune cell number was observed in the sample treatment groups compared to the LPS group, although this trend did not reach statistical significance (Fig. 4A-4D). Wright’s staining further confirmed a reduction in neutrophils in BALF following CME treatment (Fig. 4E).

### CME Inhibits the Expression of Cytokines and Chemokines Induced by LPS in RAW 264.7 Cells

**Fig. 5:**
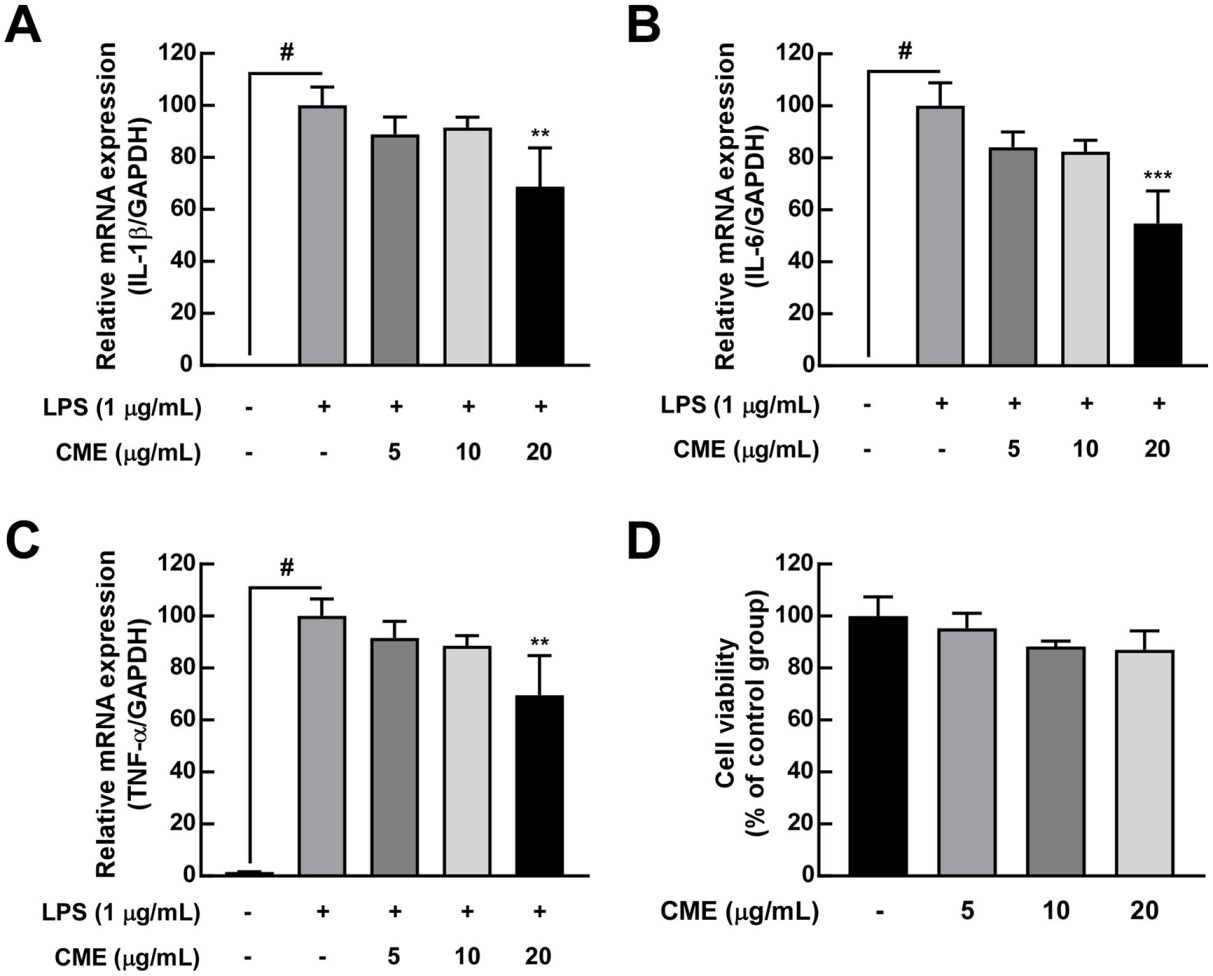
CME inhibits cytokine expression in LPS-stimulated RAW 264.7 cells. (**A**–**C**) ELISA results show reductions in IL-1β, IL-6, and TNF-α protein levels following CME treatment. (**D**) Cell viability assay confirms CME treatment is non-toxic at experimental doses. Data are presented as mean ± SD. #*p* < 0.05 for the LPS induction level; **p < 0.01, ***p < 0.001 compared to the LPS-only group.

We further evaluated the effect of CME on LPS from *K. pneumoniae*-induced inflammatory markers in RAW 264.7 murine macrophages. In RAW 264.7 cells, the mean relative mRNA expression level of IL-1β was 0.00002 in the control group, 1.00000 in the LPS group, 0.88893 in the low group, 0.91431 in the middle group, and 0.68743 in the high group. The mean IL-6 mRNA expression level was 0.00003 in the control group, 1.00000 in the LPS group, 0.84000 in the low group, 0.82392 in the middle group, and 0.54742 in the high group. The mean TNF-α expression level was 0.01571 in the control group, 1.00000 in the LPS group, 0.91550 in the low group, 0.88514 in the middle group, and 0.69579 in the high group. Stimulation with LPS resulted in increased expression of mRNA of pro-inflammatory cytokines including IL-1β, IL-6, and TNF-α. CME pretreatment significantly suppressed the mRNA levels of IL-1β, IL-6, and TNF-α (Fig. 5A-5C). Cell viability assays confirmed that CME concentrations used in the study were non-toxic (Fig. 5D).

### CME Inhibits LPS-Induced NF-κB Activation in RAW 264.7 Cells

**Fig. 6:**
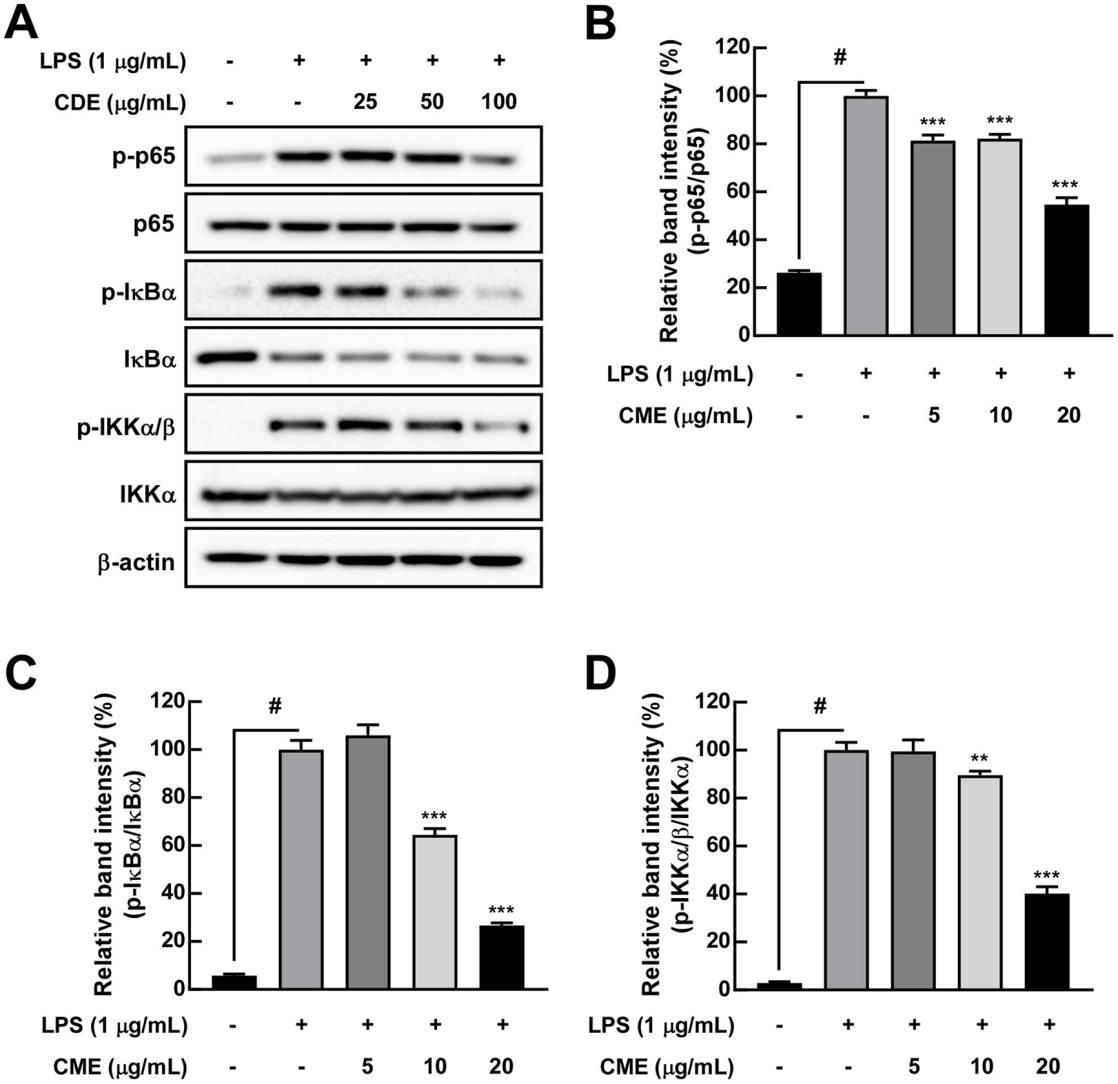
CME suppresses NF-κB pathway activation in LPS-stimulated RAW 264.7 cells. (**A**) Western blot analysis shows reduced phosphorylation of p65, IκBα, and IKKα in CME-treated cells. (**B**–**D**) Quantitative analysis of NF-κB pathway proteins. Data are presented as mean ± SD. #*p* < 0.05 for the LPS induction level; **p < 0.01, ***p < 0.001 compared to the LPS-only group.

The NF-κB signaling pathway, a critical regulator of inflammatory responses, was activated by LPS treatment, as evidenced by the phosphorylation of p65, IκBα, and IKKα [17]. Quantification of relative western blot band intensity of NF-κB signaling pathway markers in RAW 264.7 cells showed the mean phosphorylated p65 level was 26.3 in the control group, 100.0 in the LPS group, 81.4 in the low group, 82.2 in the middle group, and 64.6 in the high group. The mean phosphorylated IκB level was 5.7 in the control group, 100.0 in the LPS group, 106.0 in the low group, 64.4 in the middle group, and 26.7 in the high group. The mean phosphorylated IKK level was 2.9 in the control group, 100.0 in the LPS group, 99.5 in the low group, 89.6 in the middle group, and 40.1 in the high group. CME pretreatment significantly inhibited the phosphorylation of IκBα, IκB, and p65, indicating suppression of NF-κB pathway activation (Fig. 6A-6D).

## Discussion

Respiratory diseases including COPD and asthma are increasingly prevalent due to environmental factors such as air pollution and particulate matter exposure [18]. These diseases are characterized by chronic inflammation, tissue damage, and impaired respiratory function. The COVID-19 pandemic has further underscored the importance of identifying functional materials that support respiratory health. This study demonstrates that CME effectively mitigates LPS-induced lung injury and inflammation in both *in vivo* and *in vitro* models, highlighting its potential as a functional health supplement.

LPS-induced lung injury models mimic the inflammatory processes observed in respiratory diseases. In this study, CME suppressed immune cell infiltration, reduced collagen deposition, and alleviated mucus hypersecretion in LPS-treated mice. These findings align with previous reports on the anti-inflammatory and protective properties of *C. militaris* [19]. Notably, oral administration of CME suppressed LPS-induced immune cell infiltration and fibrosis in the mouse lungs. Additionally, CME reduced the expression of MUC5AC, a protein implicated in airway obstruction and respiratory dysfunction in diseases such as COPD and asthma [20, 21]. Wei *et al*. and Chen *et al*. reported that *C. militaris* is an excellent nutraceutical material that inhibits the infiltration of immune cells, collagen deposition, and the expression of mucin [22, 23]. Therefore, CME is also potentially an excellent nutraceutical for respiratory health, inhibiting excessive infiltration of immune cells, collagen deposition, and overexpression of MUC5AC.

Inflammatory responses in respiratory diseases, often driven by inflammatory proteins, recruit immune cells to the site of inflammation and cause tissue damage [24]. In this study, oral administration of CME significantly suppressed LPS-induced expression of iNOS and COX-2 in lung tissue and attenuated the production of pro-inflammatory cytokines (IL-1β, IL-6, and TNF-α) and the chemokine CXCL1 in the BALF of mice, indicating its potential to modulate inflammatory responses. Furthermore, LPS induced an increase in the total immune cell, neutrophil, monocyte, and eosinophil numbers in BALF, with notable recruitment of neutrophils, while CME administration tended to decrease the average cell number in the sample group compared with the LPS group. During lung inflammation, immune cell infiltration is a key pathological feature [25]. Therefore, the reduction in immune cell numbers supports the role of CME in mitigating excessive immune activation and inflammation.

The NF-κB pathway plays a pivotal role in regulating inflammatory mediators, and inhibition of NF-κB may be an important strategy for mitigating pathogen exposure-induced lung inflammation or damage [9, 26]. In RAW 264.7 macrophages, CME inhibited NF-κB activation by suppressing the phosphorylation of p65, IκBα, and IKKα. This mechanism is consistent with the observed reductions in cytokine expression and tissue damage in organisms, suggesting that CME exerts its protective effects through modulation of the NF-κB pathway.

The unique composition of *C. militaris* ARA301, particularly its high cordycepin content, may underlie its potent bioactivity. Cordycepin has been shown to exert anti-inflammatory and immunomodulatory effects by targeting signaling pathways such as NF-κB and mitogen-activated protein kinases (MAPKs) [27]. Leveraging this bioactive compound, CME offers a promising approach for preventing lung injury.

This study highlights the potential of CME to act as a functional health supplement for respiratory disease prevention by alleviating lung injury and acute inflammation in mouse lungs and BALF, respectively. However, further research is necessary to validate these findings in human clinical trials and to investigate the long-term safety and efficacy of CME consumption. Detailed characterization of the active components of CME and their mechanisms of action may also reveal additional therapeutic applications.

## Conclusion

CME effectively alleviates LPS-induced lung injury, including fibrosis and mucus deposition, by suppressing inflammatory responses, reducing immune cell recruitment and production of inflammatory proteins, and modulating the NF-κB signaling pathway. These findings suggest that CME holds significant potential as a functional material for improving respiratory health.

## Figures and Tables

**Table 1:** Primer sequences.

Species	Gene	Sense strand (5'-3')	Antisense strand (3'-5')
Mouse	IL-1β	GTT GAT GTG CTG CTG CGA GA	AGT TGA CGG ACC CCA AAA GAT
	IL-6	AGC CTC CGA CTT GTG AAG TGG T	TGG GAC TGA TGC TGG TGA CAA C
	TNF-α	AGA GGC TGA GAC ATA GGC ACC G	TGG AAC TGG CAG AAG AGG CAC T
	GAPDH	TCA ACG GCA CAG TCA AGG	ACT CCA CGA CAT ACT CAG C
